# Win–Win Pricing of Follow-Up Policies Under Healthcare Warranties for Chronic Diseases: A Mathematical Modeling Approach

**DOI:** 10.3390/healthcare13192461

**Published:** 2025-09-28

**Authors:** Mei Li, Zixian Liu, Lijun Liang

**Affiliations:** 1 School of Management, Xi’an University of Architecture and Technology, Xi’an 710055, China; limeitju@hotmail.com; 2 College of Management & Economics, Tianjin University, Tianjin 300072, China; liuzixian@tju.edu.cn; 3 School of Management, Tianjin University of Traditional Chinese Medicine, Tianjin 301617, China

**Keywords:** healthcare management, chronic disease, follow-up policy, healthcare warranty, win-win price interval, medical decision making

## Abstract

Implementing follow-up policies under healthcare warranties for chronic disease patients plays a crucial role in reducing the risk of adverse outcomes (AOs) and controlling long-term medical costs. However, the additional cost associated with these services often discourages hospitals from providing them. **Background/Objectives**: To incentivize participation from both hospitals and patients in follow-up programs, this paper introduces a patient copayment mechanism. We propose a theoretical mathematical modeling framework to investigate the optimal pricing of follow-up policies from both patients’ and hospitals’ perspectives to achieve win–win outcomes. **Methods**: Using the Cox frailty model, we stratify patients by risk level and model hazard rate functions for three follow-up policies featuring periodic checkups, incorporating the virtual age method. Building on this framework, we employ the Non-Homogeneous Poisson Process to analyze the total expected costs incurred by hospitals and patients across different policies and risk strata. This analysis derives the minimum price acceptable to hospitals for providing follow-up services and the maximum additional cost patients are willing to bear for them. The feasibility and applicability of the proposed model are demonstrated through a case study of pediatric type 1 diabetes mellitus (T1DM). **Results**: Win–win price intervals for T1DM patients are more achievable for higher-risk individuals. These intervals narrow or widen with the age reduction factor, checkup cost, and AO treatment cost. Hospitals should prioritize higher-risk patients, improve checkup effectiveness, and balance costs of checkups and treatments when optimizing pricing decisions. **Conclusions**: These insights provide valuable guidance for hospitals in strategically designing follow-up policies tailored to diverse risk cohorts and determining optimal price intervals.

## 1. Introduction

Chronic diseases, such as diabetes mellitus and hypertension, represent a major threat to public health and socioeconomic development globally. According to data from the World Health Organization, approximately 41 million people die from noncommunicable chronic diseases annually, accounting for 74% of global deaths. Characterized by prolonged duration and persistent progression, these conditions entail a high risk of developing a number of adverse outcomes (AOs), such as disease recurrence and complications, significantly impairing patients’ quality of life while imposing substantial cost burdens on both individuals and the whole healthcare system.

A follow-up policy, comprising scheduled checkups during a defined post-discharge period, has proven effective in delaying chronic disease progression and preventing AOs, thereby controlling medical costs. In practice, however, most hospitals do not implement structured follow-up policies, as they face no mandate to do so and would incur additional costs. Typically, patients are advised to conduct a follow-up based on clinical practice guidelines and their providers’ clinical experience. This ad hoc approach often lacks continuity, potentially leading to increased AOs and associated costs. In an effort to contain healthcare expenditures, the United States enacted the Patient Protection and Affordable Care Act in 2010. This legislation spurred new payment programs designed to replace the traditional fee-for-service (FFS) model that has long been criticized for lacking preventive care incentives. Among these innovations, healthcare warranties (inspired by product warranty concepts [1,2]) have gained significant attention due to their effectiveness. Under such a warranty, hospitals receive a bundled payment covering all services during initial hospitalization and a predefined post-discharge warranty period, regardless of the actual cost incurred by patients [3,4]. If a patient experiences an AO during the warranty period, the hospital will deliver treatment at no additional cost to the patient. By transferring the financial risk from patients to providers, warranties incentivize hospitals to proactively implement continuous follow-up policies to mitigate AO risks and costs. At present, healthcare warranties are applied not only in specific organizations, such as ProvenCare in Geisinger [5] and PROMETHEUS [6], but also nationwide, such as in the Bundled Payments for Care Improvement (BPCI) and BPCI advanced models proposed by the Centers for Medicare & Medicaid Services (CMS) [7,8]. Similar practices have emerged in other countries. For instance, in China’s Anhui Province, public hospitals began to implement healthcare warranties for patients with appendicitis in 2011, adopting a policy that bundles payment for the initial admission and any readmission within 15 days after discharge [9]. Similarly, in Germany, hospitals receive a lump-sum payment covering the initial treatment and any readmissions occurring within a certain time period [10]. The chronic diseases involved in warranty practices include diabetes mellitus, cardiovascular disease, chronic obstructive pulmonary disease, etc. [11,12,13].

However, some hospitals contend that they are penalized unfairly by the healthcare warranty reimbursement scheme, since the AOs occurrence depends heavily on patient behavior, which is not controllable by the hospitals [14]. Effective disease management indeed necessitates collaborative efforts between hospitals and patients to achieve optimal outcomes. The successful delay and control of disease progression largely depends on patients’ self-management practices, such as lifestyle modifications and regular monitoring of relevant health indicators [15,16]. In such situations, even well-designed follow-up policies fail to deliver value without patient adherence to scheduled protocols. Implementing cost-sharing for these policies serves a dual purpose: motivating proactive disease management while ensuring compliance, thereby guaranteeing intervention effectiveness. This interdependence makes pricing decisions for follow-up policies strategically crucial. Elevated pricing discourages patient participation, whereas insufficient pricing risks hospital losses and service discontinuation. Therefore, it is necessary to study the pricing of follow-up policies under healthcare warranties from the perspectives of both patients and hospitals to achieve win–win outcomes.

Our work is relevant to two streams of research: (i) follow-up policy optimization, (ii) healthcare warranty pricing.

Follow-up policy optimization. There has been a growing body of research on follow-up policy design and optimization for chronic disease. Helm et al. [17] optimized the timing of the next follow-up based on a current visit for glaucoma patients through an integrated model of Kalman filtering and mathematical programming. Based on this work, Kazemian et al. [18] developed a linear quadratic Gaussian state space model to simultaneously optimize follow-up timing and modality, as well as control thresholds for key risk indicators. Some research utilized Markov decision processes (MDPs) to investigate sequential follow-up policies involving decisions at predetermined intervals [19,20]. Additional studies optimized finite-horizon follow-up policies, under which follow-up timing and modalities over a specified period are determined simultaneously, focusing either on maximizing the policy’s detection rate for potential AOs [21] or enhancing the checkup’s ability to reduce AO risk [22,23]. The aforementioned studies lay the analytical foundation for our work on follow-up policy cost and associated healthcare warranty cost estimation. However, while these studies aim to optimize the timing and modalities of a follow-up policy, our research focuses on determining the win–win interval price for follow-up policies based on relevant cost analysis.

Healthcare warranty pricing. BP practices typically determine package prices based on the average historical cost of providing the services. For example, ProvenCare’s case rate for CABG patients encompasses not only the historical average cost of all follow-up care and rehospitalizations for postoperative complications within 90 days of surgery, but also approximately 50% of the mean cost of all related postoperative readmissions derived from a two-year historical financial comparison group [24]. Similarly, PROMETHEUS proposes evidence-informed case rates comprising three cost components: base services, severity adjusters, and potentially avoidable complications—all calculated from historical data [25]. The BPCI sets episode prices based on a discounted aggregation of historical payments for services preciously rendered during such episodes [26]. However, these retrospective pricing approaches fail to account for the efficiency or effectiveness of service delivery. To address this limitation, recent research has developed alternative pricing models. For instance, Hellsten et al. developed innovative clinically informed pricing models for BP by leveraging clinical standards and regional variations, using Ontario stroke care as a case study [27]. Cher et al. [28] devised an empirical Bayes approach to calculate target prices for BPCI, while Tanwar et al. [29] established an efficient pricing interval, bounded by profit maximization and risk minimization values, for fixed price contracts.

Crucially, existing research relies on current healthcare warranty practices, wherein follow-up services and other necessary care are bundled. When pricing such packages, the cost of complications or readmissions receives significant attention, whereas follow-up costs are overlooked. Yet follow-up expenses depend on diverse factors: the frequency and efficacy of checkups, patient risk levels, and other variables. Consequently, follow-up costs can constitute a substantial portion of warranty pricing. Ignoring these costs may discourage patients from proactively managing their conditions. Furthermore, current studies adopt singular perspectives, either the payer’s or the hospital’s, neglecting patient considerations. An equitable price must balance the needs of both patients and hospitals. It should encourage patients’ willingness to pay for follow-up services while ensuring hospitals’ acceptance of their delivery.

To bridge these gaps, this paper proposes a theoretical mathematical modeling framework to investigate the pricing of follow-up policies for chronic diseases under healthcare warranties from dual stakeholder perspectives (hospitals and patients) to achieve mutually beneficial outcomes. The primary purpose is to determine whether there exists a pricing zone of follow-up policies acceptable for both hospitals and patients, thereby establishing a win–win relationship between the two parties. We model heterogeneous patients with varying AO risk levels and three follow-up policies differentiated by implementation timeframes. Hospitals set prices for each policy, and patients select policies based on these prices. By analyzing hazard rate functions and associated costs across policies and risk levels, we derive win–win price intervals for follow-up services under diverse scenarios.

The remainder of this paper is organized as follows. Section 2 presents the problem description, necessary assumptions and notations, and formulates the mathematical model for pricing of follow-up policies. In Section 3, we present a case study of pediatric type 1 diabetes mellitus and conduct sensitivity analyses of key parameters. Section 4 discusses the principal findings, practical implications, limitations, and potential future research extensions. Conclusions are presented in Section 5.

## 2. Materials and Methods

### 2.1. Problem Description

Consider a healthcare delivery system involving two parties, i.e., a hospital and its cohort of patients diagnosed with a specific chronic disease, such as diabetes mellitus. The hospital provides these patients with a free healthcare warranty, which is a common practice currently implemented in programs such as PROMETHEUS [6] and ProvenCare [5]. Within the warranty period W, all AOs are treated by the hospital free of charge, while after the warranty expires, patients have to bear the treatment cost. To mitigate the financial risk associated with providing the healthcare warranty, the hospital simultaneously offers post-discharge follow-up policies, denoted as $\left[n,\tau \right]$, within the planning horizon $L\;\left(L>W\right)$. The planning horizon represents a fixed period during which the components of implemented follow-up policies, such as frequency and intervention, remain unchanged. However, from a long-term perspective, hospitals must dynamically adjust policy configurations to adapt to patients’ evolving disease progression. These policies, offered for a fee, enable disease progression monitoring and timely intervention to avoid and prevent AOs. n and τ represent the number of checkups and the time interval between two consecutive checkups, respectively. Most studies on follow-up policy optimization focus on periodic policies due to their implementational simplicity [22,30]. Consequently, we assume in this paper the hospital offers periodic follow-up policies wherein checkups occur at uniform time intervals. Patients may consider purchasing follow-up services at a reasonable price to reduce potential treatment costs after warranty expiry. Conversely, the hospital needs to avoid losses from providing follow-up services. This situation creates a decision-making challenge for both parties. The objective of our research is to discover, under different follow-up policies, if there exists a pricing zone of follow-up service acceptable for both hospitals and patients. In this study, we consider the following follow-up policies.

- Follow-up policy I: periodic checkups within the warranty period $\left[0\right.,\left.W\right)$.
- Follow-up policy II: periodic checkups within the post-warranty period $\left[W\right.,\left.L\right)$.
- Follow-up policy III: periodic checkups within the planning horizon $\left[0\right.,\left.L\right)$.

**Figure 1:**
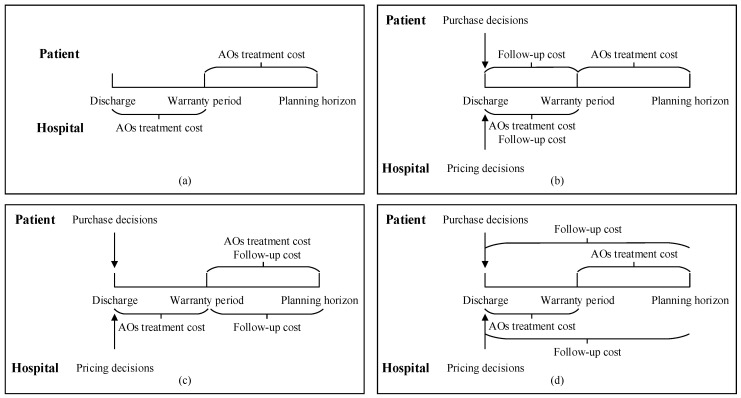
Costs incurred by hospitals and patients under different follow-up policies. (**a**) No follow-up services; (**b**) follow-up policy I; (**c**) follow-up policy II; (**d**) follow-up policy III.

In the absence of follow-up services, the hospital is only responsible for the AO treatment costs that occur during the warranty period, with patients bearing those within the post-warranty period. When any of the three follow-up policies is implemented, both the hospital and the patient bear the expenses for follow-ups and AO treatments during their respective timeframes. The policy pricing decisions for the hospital and the policy purchase decisions for the patient at discharge will be based on the expected values of these costs. Costs incurred by hospitals and patients under different policies are presented in Figure 1.

The following assumptions and notation (shown in Table 1) are needed to facilitate the model formulation.

**Model assumptions**:

- The hazard rate function of time to develop an AO after discharge increases monotonically over time. Moreover, for model tractability, we assume the hazard rate at discharge is zero, which is in accordance with studies discussing follow-up policies [31,32]. The justification for this assumption is threefold. First, patients are discharged only when they are clinically stable and free from any uncontrolled or potential AOs. Second, discharge represents the starting point of our study, which is an instantaneous point in time rather than a duration. Since patients are discharged in a stable condition, the hazard rate (i.e., the immediate risk of developing AOs) can be considered zero at this moment and will increase thereafter. For instance, pediatric type 1 diabetes patients face a high risk of developing ketoacidosis and hypoglycemia. These AOs can be cured through hospitalization and treatment, and the hazard rate at discharge can be regarded as zero. Subsequently, inadequate glucose monitoring, improper insulin administration, and suboptimal dietary management may gradually elevate the hazard rate. Third, our primary focus lies on the evolution of risk after discharge rather than the state at the point of discharge itself. Additionally, Tarakci et al. have demonstrated that follow-up services are unnecessary for monotonically decreasing hazard rates, while the optimal interval approaches infinity for constant hazard rates [32].
- Relative to the time to develop an AO, both AO treatment and follow-up implementation durations are negligible, which is consistent with the work by Bavafa et al. [31] and Li et al. [22].
- During the warranty period, the treatment will not change the trend of the hazard rate function of the time to develop an AO [32,33,34]. This assumption conceptualizes AO treatment as a minimal repair in the machine maintenance literature. In such contexts, minimal repair is standardly assumed for systems with multiple components or failure modes. All failures have been rectified through minimal repair when the failed item is restored to an operational state, but its failure rate remains unchanged [35,36]. Given that healthcare patients face multiple risk factors and various types of AOs, analogous to multi-failure-mode systems, we adopt this consistent theoretical framework.

**Model notation**:

**Table 1:** Model notation.

Notation	Description
N	Number of patient risk groups
$P_{k}$	Price of follow-up policy for the kth risk group, k=1,2,⋯,N
n	Number of checkups in a follow-up policy
τ	Time interval between two consecutive checkups in a follow-up policy
$n_{j}$	Number of checkups within a time horizon, j=1,2,3,4
$t_{i}$	Timing of the ith checkup, i=1,2,⋯,n
W	Length of the warranty period
L	Length of the planning horizon
X	Vector of observable patient heterogeneity
B	Vector of coefficients associated with X
Z	Random variable for unobservable patient heterogeneity
h	Random variable for patient heterogeneity, $h=Ze^{XB}$
$\left[h_{k-1},h_{k}\right)$	Interval of h for the kth risk group
$G\left(h\right),g\left(h\right)$	CDF and PDF of h
T	Random variable for the time-to-develop an AO
$\lambda_{0}\left(t\right)$	Baseline hazard rate function of T
$F\left(t\right),f\left(t\right)$	CDF and PDF of T
$\lambda \left(t|h\right)$	Conditional hazard rate function of T under h
θ	Age reduction factor of a follow-up checkup
$c_{f}$	Average cost of implementing a follow-up checkup
$c_{kr}$	Average cost of treating an AO for the kth risk group

### 2.2. Model Formulation

#### 2.2.1. Modeling the Time to Develop an AO

In the medical field, survival analysis methods are widely used to fit the probability distribution of time to an event, such as the Kaplan–Meier method [37], the Cox proportional hazards model [38], and the Cox frailty model [39]. Among these, the Cox frailty model offers distinct advantages over other methods, as it can simultaneously account for the influence of both observed heterogeneity (such as individual characteristics like age and sex, as well as clinical features like disease severity) and unobserved heterogeneity (such as adherence to medical advice) on the time to develop an AO [40,41].

Consider a patient with a certain chronic disease who, after discharge, develops a disease-related AO at time T, requiring them to seek medical attention again. It is noted that T<W, where W is the length of the healthcare warranty period. Let $F\left(t\right)$ and $f\left(t\right)$ denote the cumulative distribution function (CDF) and probability density function (PDF) of T, respectively. Then, the baseline hazard rate function $\lambda_{0}\left(t\right)$ for T can be expressed as follows:

(1) $$\lambda_{0}\left(t\right)=\frac{f\left(t\right)}{1-F\left(t\right)},$$

where t≥0. According to the first assumption, $\lambda_{0}\left(0\right)=0,\;\lambda_{0}^{\prime }\left(t\right)>0$. The baseline hazard rate function is assumed to be the same form for all individuals in the patient population but is modified to account for patient heterogeneity. Specifically, to incorporate both observable and unobservable patient heterogeneity, we employ the Cox frailty model. This model extends the standard Cox proportional hazard model by introducing a frailty factor that acts multiplicatively on the baseline hazard function, expressed as:

(2) $$\lambda \left(t|X,Z\right)=Z\lambda_{0}\left(t\right)e^{XB},$$

where Z is a positive random variable which represents the frailty factor, capturing the effect of unobservable heterogeneity on the hazard function. X is a vector of covariates reflecting observable heterogeneity, and B is the corresponding vector of coefficients. The parameters in B can be estimated from the observed data. Equation (2) reveals that the effects of observable heterogeneity $e^{XB}$ and unobservable heterogeneity Z combine multiplicatively to influence the hazard function. This combined heterogeneity effect can be represented by a single random variable. We therefore define $h=h\left(X,Z\right)=Ze^{XB}$ as this combined effect variable, whose distribution characterizes patient heterogeneity. Consequently, Equation (2) becomes

(3) $$\lambda \left(t|h\right)=h\lambda_{0}\left(t\right).$$

It is evident that a higher value of h corresponds to a greater value of $\lambda \left(t|h\right)$. Therefore, patients can be stratified into different risk groups based on their h values, enabling the implementation of tailored follow-up strategies for each group. This customized approach facilitates improved patient management and cost containment for hospitals. The stratification method can be given as follows:

(4) $$\int_{h_{k-1}}^{h_{k}}g\left(h\right)dh=\frac{1}{N}\int_{\underline{h}}^{\bar{h}}g\left(h\right)dh,\;k=1,\;2,\;\cdots ,\;N,$$

(5) $$h_{0}=\underline{h},\;h_{N}=\bar{h},$$

where N is the number of risk groups in the patient population. The kth group corresponds to the interval $\left[h_{k-1},h_{k}\right)$. The probability distribution function of h is denoted by $g\left(h\right)$, with $\underline{h}$ and $\bar{h}$ representing its minimum and maximum observed values, respectively. The stratification method partitions patients uniformly across the range of h into N groups, facilitating subsequent comparison of group-specific metrics.

#### 2.2.2. Modeling the Effect of Follow-Up Checkups

In practice, a follow-up checkup typically comprises treatment adjustment, diet and lifestyle advice, and other interventions. Following such a checkup, the risk of developing an AO is effectively reduced. Thus, modeling the impact of follow-up checkups on the hazard rate function is essential. To achieve this, we employ the virtual age method. This approach, based on the premise that each checkup rejuvenates the patient’s health state (thus lowering their future hazard rate), has been applied in many follow-up policy studies [22,23].

Let $v_{i-1}$ be the virtual age of a patient after the $\left(i-1\right)$th checkup. Then, the virtual age after the ith checkup can be given by

(6) $$v_{i}=v_{i-1}+\tau \theta =i\tau \theta ,\;i=1,2,\cdots ,n,$$

where $v_{0}=0$. τ is the time interval between two consecutive checkups within a follow-up policy. θ is the age reduction factor of a follow-up checkup, representing the effectiveness to reduce AO risk. A smaller θ value corresponds to greater risk reduction efficacy.

**Figure 2:**
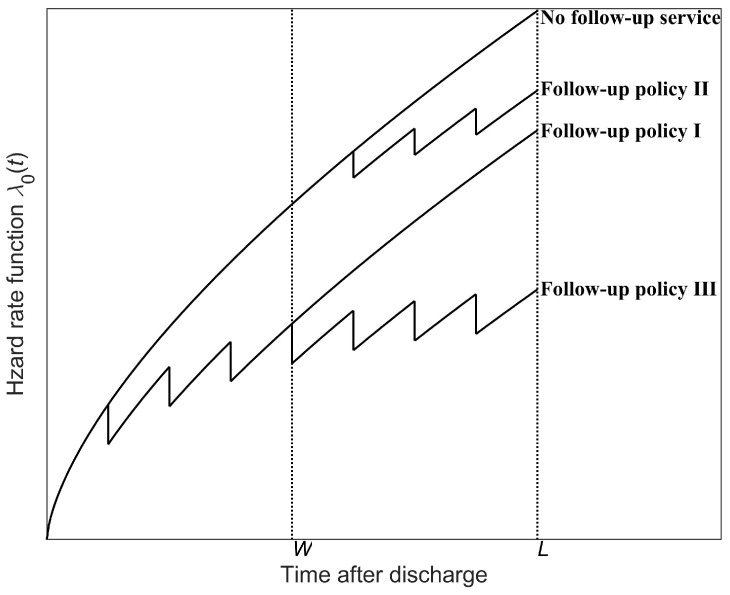
A comparison of hazard rate functions under different follow-up policies.

Figure 2 presents a comparison of the hazard rate under different follow-up policies using the virtual age method. It can be seen that when no follow-up service is provided, the risk of developing an AO continues to rise over time and remains consistently higher than in cases with follow-up services. When hospitals offer follow-up services, the AO risk decreases after each follow-up checkup. Among different follow-up policies, follow-up policy III results in the lowest AO risk for patients, with policy II presenting a higher risk than policy I.

Generally, the hazard rate function can be transformed into a form expressed through virtual age, i.e., $\lambda \left(v_{i}+t|h\right)$ for $t\in \left[0,\tau )\right.$ or $\lambda \left(v_{i}+t-t_{i}|h\right)$ for $t\in \left[t_{i},t_{i+1})\right.$, where $t_{i}$ denotes the timing of the ith checkup. Consequently, hazard rate functions over $\left[0\right.,\left.W\right)$ and $\left[W\right.,\left.L\right)$ under different follow-up policies are summarized in Table 2. Definitions and calculations for the number of checkups within a time horizon $n_{j},j=1,2,3,4$ are provided in Table 3.

#### 2.2.3. Cost Functions for Patients and Hospitals

Let $C_{pn}^{X}$ denote the total expected cost incurred when a patient does not purchase follow-up services within the planning horizon L, where X=I,II,III. In this scenario, the patient bears the treatment cost for AOs occurring in the interval $\left[W\right.,\left.L\right)$. This cost equals the product of the expected number of AOs in this interval and the average treatment cost per AO. Assuming AO arrivals follow a Non-Homogeneous Poisson Process (NHPP), the treatment cost for a patient in the kth risk group is given by:

(7) $$C_{pn}^{X}=c_{kr}\int_{h_{k-1}}^{h_{k}}\int_{W}^{L}\lambda \left(t|h\right)dtdG\left(h\right),$$

where $c_{kr}$ is the average cost of treating an AO for a patient in the kth risk group, and $G\left(h\right)$ is the cumulative distribution function of h.

Let $C_{hn}^{X}$ denote the total expected cost incurred by the hospital when it does not provide follow-up services within the planning horizon L, where X=I,II,III. In this case, the hospital only incurs AO treatment costs within the warranty period:

(8) $$C_{hn}^{X}=c_{kr}\int_{h_{k-1}}^{h_{k}}\int_{0}^{W}\lambda \left(t|h\right)dtdG\left(h\right).$$

Let $C_{py}^{X}$ denote the total cost incurred when a patient purchases follow-up services, and $C_{hy}^{X}$ the total cost incurred by the hospital when providing these services within the planning horizon L, where X=I,II,III. If a patient purchases the follow-up services, they must incur both treatment costs for AOs during $\left[W\right.,\left.L\right)$ and follow-up costs. Meanwhile, hospitals providing these services face costs comprising both treatment costs for AOs within the warranty period and implementation costs for follow-up checkups. Since different follow-up policies involve distinct numbers of checkups and corresponding hazard rate functions, these costs consequently vary across policies and will be analyzed separately below.

- **Follow-up policy I: periodic checkups under warranty.**

Under this policy, the hospital carries out follow-up checkups within the warranty period W and the number of checkups is set as $n_{1}$. During the planning horizon, the cost borne by the patient in the kth risk group when purchasing follow-up policy I is

(9) $$C_{py}^{I}=P_{k}+c_{kr}\int_{h_{k-1}}^{h_{k}}\int_{W}^{L}\lambda_{PW}^{I}\left(t|h\right)dtdG\left(h\right),$$

where $P_{k}$ denotes the fee paid by the patient in the kth risk group for follow-up services. The cost incurred to the hospital when providing follow-up policy I for a patient in the kth risk group is

(10) $$C_{hy}^{I}=c_{kr}\int_{h_{k-1}}^{h_{k}}\int_{0}^{W}\lambda_{W}^{I}\left(t|h\right)dtdG\left(h\right)+n_{1}c_{f}-P_{k}.$$

- **Follow-up policy II: periodic checkups during the post-warranty period.**

Under this policy, follow-up checkups are implemented from the end of the warranty $\left[W\right.,\left.L\right)$, and the number of checkups is set as $n_{2}$. Thus, during the planning horizon, the cost borne by the patient in the kth risk group when purchasing follow-up policy II is

(11) $$C_{py}^{II}=P_{k}+c_{kr}\int_{h_{k-1}}^{h_{k}}\int_{W}^{L}\lambda_{PW}^{II}\left(t|h\right)dtdG\left(h\right).$$

The cost incurred to the hospital when providing follow-up policy II for a patient in the kth risk group is

(12) $$C_{hy}^{II}=c_{kr}\int_{h_{k-1}}^{h_{k}}\int_{0}^{W}\lambda_{W}^{II}\left(t|h\right)dtdG\left(h\right)+n_{2}c_{f}-P_{k}.$$

- **Follow-up policy III: periodic checkups over the planning horizon.**

For this policy, the hospital performs follow-up checkups over the planning horizon $\left[0\right.,\left.L\right)$, and the numbers of checkups within $\left(0\right.,\left.L\right]$ and $\left(0\right.,\left.W\right]$ are set as $n_{3}$ and $n_{4}$, respectively. Then, the cost borne by the patient in the kth risk group when purchasing follow-up policy III is

(13) $$C_{py}^{III}=P_{k}+c_{kr}\int_{h_{k-1}}^{h_{k}}\int_{W}^{L}\lambda_{PW}^{III}\left(t|h\right)dtdG\left(h\right).$$

The cost incurred to the hospital when providing follow-up policy III for a patient in the kth risk group is

(14) $$C_{hy}^{III}=c_{kr}\int_{h_{k-1}}^{h_{k}}\int_{0}^{W}\lambda_{W}^{III}\left(t|h\right)dtdG\left(h\right)+n_{3}c_{f}-P_{k}.$$

#### 2.2.4. Win–Win Price Intervals for Follow-Up Policies

For patients, purchasing follow-up services incurs an additional fee but reduces both AO risk and associated treatment expenses, potentially lowering net costs compared to non-purchase. For hospitals, revenue from follow-up services offsets part of the treatment costs, creating potential profit. Therefore, follow-up service pricing must balance both stakeholder perspectives. Specifically, patients purchase services if and only if $C_{py}^{X}\leq C_{pn}^{X}$, whereas hospitals provide services if and only if $C_{hy}^{X}\leq C_{hn}^{X}$. When both conditions hold, neither party incurs net losses.

Our goal is to identify win–win price intervals for different follow-up policies. The interval’s upper limit represents the maximum price patients will pay, while the lower limit reflects the minimum price hospitals will accept. At any price within the interval, patients are willing to purchase services and hospitals are willing to provide services, enabling optimal policy.

- **Follow-up policy I: periodic checkups under warranty.**

According to the policy purchase condition $C_{py}^{I}\leq C_{pn}^{I}$, the maximum price patients will pay for follow-up policy I is

(15) $$P_{p}^{I}=c_{kr}\phi \left(h_{k}\right)\int_{W}^{L}\left[\lambda_{0}\left(t\right)-\lambda_{0}\left(v_{n_{1}}+t-t_{n_{1}}\right)\right]dt,$$

where $\phi \left(h_{k}\right)=\int_{h_{k-1}}^{h_{k}}hg\left(h\right)dh$. The proof of Equation (15) is presented in Appendix A.1.

Similarly, the policy provision condition $C_{hy}^{I}\leq C_{hn}^{I}$ gives the minimum price hospitals will accept for follow-up policy I:

(16) $$P_{h}^{I}=c_{kr}\phi \left(h_{k}\right)\left[\sum_{i=1}^{n_{1}}\int_{t_{i-1}}^{t_{i}}\lambda_{0}\left(v_{i-1}+t-t_{i-1}\right)dt+\int_{t_{n_{1}}}^{W}\lambda_{0}\left(v_{n_{1}}+t-t_{n_{1}}\right)dt-\int_{0}^{W}\lambda_{0}\left(t\right)dt\right]+n_{1}c_{f}.$$

This implies that patients decline follow-up services when $P_{k}>P_{p}^{I}$, and hospitals withhold these services when $P_{k}<P_{h}^{I}$. Therefore, the win–win price interval for follow-up policy I is $\left[P_{h}^{I},P_{p}^{I}\right]$, which exists if and only if $P_{p}^{I}>P_{h}^{I}$. The proof of Equation (16) is presented in Appendix A.2.

- **Follow-up policy II: periodic checkups during the post-warranty period.**

According to the policy purchase condition $C_{py}^{II}\leq C_{pn}^{II}$, the maximum price patients will pay for follow-up policy II is

(17) $$P_{p}^{II}=c_{kr}\phi \left(h_{k}\right)\left[\int_{t_{1}}^{L}\lambda_{0}\left(t\right)dt-\sum_{i=2}^{n_{2}}\int_{t_{i-1}}^{t_{i}}\lambda_{0}\left(v_{i-1}+t-t_{i-1}\right)dt-\int_{t_{n_{2}}}^{L}\lambda_{0}\left(v_{n_{2}}+t-t_{n_{2}}\right)dt\right].$$

According to the policy provision condition $C_{hy}^{II}\leq C_{hn}^{II}$, the minimum price hospitals will accept for follow-up policy II is

(18) $$P_{h}^{II}=n_{2}c_{f}.$$

Therefore, the win–win price interval for follow-up policy II is $\left[P_{h}^{II},P_{p}^{II}\right]$, which exists if and only if $P_{p}^{II}>P_{h}^{II}$. The proof of Equations (17) and (18) is similar to that of Equations (15) and (16) and is thus omitted.

- **Follow-up policy III: periodic checkups over the planning horizon.**

According to the policy purchase condition $C_{py}^{III}\leq C_{pn}^{III}$, the maximum price patients will pay for follow-up policy III is

(19) $$P_{p}^{III}=c_{kr}\phi \left(h_{k}\right)\left[\int_{W}^{L}\lambda_{0}\left(t\right)dt-\int_{W}^{t_{n_{4}+1}}\lambda_{0}\left(v_{n_{4}}+t-t_{n_{4}}\right)dt-\sum_{i=n_{4}+1}^{n_{3}}\int_{t_{i-1}}^{t_{i}}\lambda_{0}\left(v_{i-1}+t-t_{i-1}\right)dt-\int_{t_{n_{3}}}^{L}\lambda_{0}\left(v_{n_{3}}+t-t_{n_{3}}\right)dt\right].$$

According to the policy provision condition $C_{hy}^{III}\leq C_{hn}^{III}$, the minimum price hospitals will accept for follow-up policy III is

(20) $$P_{h}^{III}=c_{kr}\phi \left(h_{k}\right)\left[\sum_{i=1}^{n_{4}}\int_{t_{i-1}}^{t_{i}}\lambda_{0}\left(v_{i-1}+t-t_{i-1}\right)dt+\int_{t_{n_{4}}}^{W}\lambda_{0}\left(v_{n_{4}}+t-t_{n_{4}}\right)dt-\int_{0}^{W}\lambda_{0}\left(t\right)dt\right]+n_{3}c_{f}.$$

Therefore, the win–win price interval for follow-up policy II is $\left[P_{h}^{III},P_{p}^{III}\right]$, which exists if and only if $P_{p}^{III}>P_{h}^{III}$. The proof of Equations (19) and (20) is similar to that of Equations (15) and (16) and is thus omitted.

## 3. Results

In this section, pediatric type 1 diabetes mellitus (T1DM) is taken as an example to verify the feasibility and effectiveness of the proposed model. T1DM is associated with multiple risk factors that predispose individuals to acute AOs, including ketoacidosis and hypoglycemia, as well as chronic AOs such as nephropathy and retinopathy. Currently, there are already some healthcare warranty practices for diabetes mellitus, such as PROMETHEUS [42]. Effective T1DM management not only requires hospitals to develop rational treatment regimens but also necessitates long-term follow-up policies. During each follow-up checkup, activities such as treatment plan adjustments, patient education, and indicator monitoring are conducted. To ensure hospitals are willing to provide these follow-up policies and patients are willing to purchase them, an appropriate win–win price interval must be established that prevents financial losses for both parties.

### 3.1. Model Parametrization

In this paper, the research data were obtained from the endocrinology department of a children’s hospital in Tianjin, China. The hospital lacks well-established follow-up mechanisms and pricing strategies. Typically, healthcare providers empirically recommend outpatient visits every six months or annually. This has resulted in a relatively low follow-up rate among patients and suboptimal disease management outcomes. Charging a certain fee for follow-up services could effectively enhance both provider and patient engagement in the follow-up, thereby improving treatment efficacy and helping to curb healthcare costs.

A total of 143 patients with T1DM and their relevant data from 2014 to 2018 were collected. The dataset comprises two parts: one is AO treatment data, including patient demographics, family history of diabetes, comorbidities, body mass index (BMI), treatment dates, and treatment costs; the other is follow-up data, including follow-up dates, follow-up costs, and follow-up methods. Patient inclusion/exclusion criteria were applied as follows. Only patients diagnosed with T1DM as their primary condition were included in subsequent analyses, as risk factors influencing AO risk vary across different diseases. Consequently, those with T2DM or other specific types of diabetes were excluded. We also excluded patients with a first-time diagnosis of T1DM due to insufficient follow-up and treatment data required for model parametrization. Furthermore, records with missing essential information (e.g., sex) were removed. Patients with long-term complications (e.g., diabetic retinopathy) were excluded as well, since their management requires collaborative care among multiple departments. For data cleaning, variables with more than 20% missing values were excluded from the analysis. For variables with less than 20% missingness, mean imputation was applied. Physiological outliers were reviewed and either corrected or set as missing if identified as recording errors. As a result, 67 AO treatment records and 149 follow-up records from 37 patients were used for subsequent analysis.

**Figure 3:**
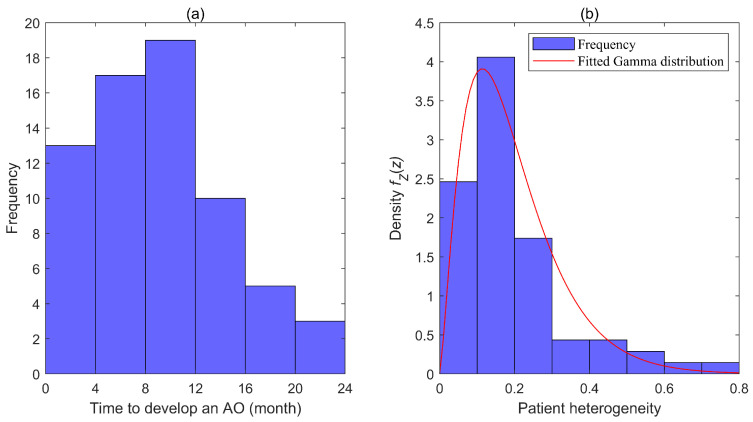
(**a**) Histogram of time to develop an AO; (**b**) Histogram and fitted Gamma distribution of patient heterogeneity.

AO treatment data are used to estimate parameters in the conditional hazard rate function: $\lambda \left(t|X,Z\right)=Z\lambda_{0}\left(t\right)e^{XB}$. Typically, the frailty factor Z is assumed to follow a Gamma distribution with a mean of 1 [43]. Consequently, the probability density function of Z is $f_{Z}\left(z\right)=\frac{z^{\frac{1}{\sigma }-1}}{\sigma^{\frac{1}{\sigma }}\Gamma \left(\frac{1}{\sigma }\right)}e^{-\frac{z}{\sigma }}$, where σ represents the variance. According to the literature and expert opinions, the observable patient heterogeneity vector X includes five components: age, gender, puberty status, number of comorbidities, length of stay, and body mass index [44,45]. Figure 3a presents the histogram of time to develop an AO. Based on that, Weibull, Gamma, and Log-Normal distributions were selected as possible candidates for the baseline hazard rate function $\lambda_{0}\left(t\right)$. We employed maximum marginal likelihood estimation to estimate the parameters, including σ and B, and the parameters of the chosen distribution for $\lambda_{0}\left(t\right)$, across different combinations of $\lambda_{0}\left(t\right)$ and $f_{Z}\left(z\right)$. The optimal results are as follows: $\lambda_{0}\left(t\right)$ follows a Weibull distribution, with the shape and scale parameters being 0.12 and 1.657, respectively; the variance of Z is 0.626; $B=\left[0.31,-0.054,0.891,-0.001,-0.083\right]$. Based on these results, we further determined that $h=Ze^{XB}$ also follows a Gamma distribution, with a mean of 2.4006 and a variance of 0.0813. Figure 3b illustrates the histogram and fitted Gamma distribution of h. Based on aforementioned results, the value of h for each patient can be calculated using the formula $h=Ze^{XB}$, along with the minimum and maximum values ($\underline{h}$ and $\bar{h}$). In this paper, patients are categorized into three risk groups: the low-, medium-, and high-risk groups. Thus, the value of N is set to 3. According to Equations (4) and (5), we have $\int_{h_{0}}^{h_{1}}g\left(h\right)dh=\int_{h_{1}}^{h_{2}}g\left(h\right)dh=\int_{h_{2}}^{h_{3}}g\left(h\right)dh=\frac{1}{3}\int_{\underline{h}}^{\bar{h}}g\left(h\right)dh$, where $h_{0}=\underline{h}$ and $h_{3}=\bar{h}$. The cut-offs for patient stratification $\left[h_{k-1},h_{k}\right)$ can therefore be determined by solving these equations. The average cost of treating an AO $c_{kr}$ for each group is defined as the mean treatment cost for AOs. The corresponding results are presented in Table 4.

The average cost of implementing a follow-up checkup $c_{f}$ is set as the mean value of follow-up cost, i.e., $c_{f}=428$. The age reduction factor θ can be estimated using a regression analysis based on changes in key patient indicators, such as blood glucose, glycosylated hemoglobin, and patient compliance with treatment, before and after checkups. However, due to data limitations, this paper is unable to obtain accurate values for θ, so we consulted several experienced endocrinologists specializing in pediatric T1DM at our partner hospital for approximate values. θ indicates the effectiveness of the follow-up checkup, and the endocrinologists believe that their follow-ups are reliable enough to detect potential AOs and reduce AO risks. Since θ∈[0,1], they all set θ=0. In order to analyze the effect of θ on AO risks, we set θ=0.1 initially and conducted a sensitivity analysis in Section 3.3.1. To facilitate comparisons between patient groups, this study assigns the same warranty period and planning horizon to all patients. The warranty period W is set to one year following several relevant studies on follow-up and healthcare warranty policy optimization for chronic disease [21,22,46]. The planning horizon L represents a fixed period during which the components of follow-up policies, such as frequency and intervention, remain unchanged. However, our partner hospital empirically recommends outpatient follow-ups every six months or annually without further adjustment. Therefore, the exact value of L cannot be directly determined from current practices. To address this, we again sought expert opinions from endocrinologists specializing in pediatric T1DM at our partner hospital. They suggested that L be defined as the timeframe during which patients remain free of long-term complications, since major adjustments to follow-up policies are typically required once complications emerge. According to clinical practice guidelines [47,48], patients often develop long-term complications within two to five years after diabetes onset. Thus, we initially set L as three years and performed a sensitivity analysis in Section 3.3.4. The values of the relevant parameters are summarized in Table 5.

### 3.2. Win–Win Price Intervals

**Figure 4:**
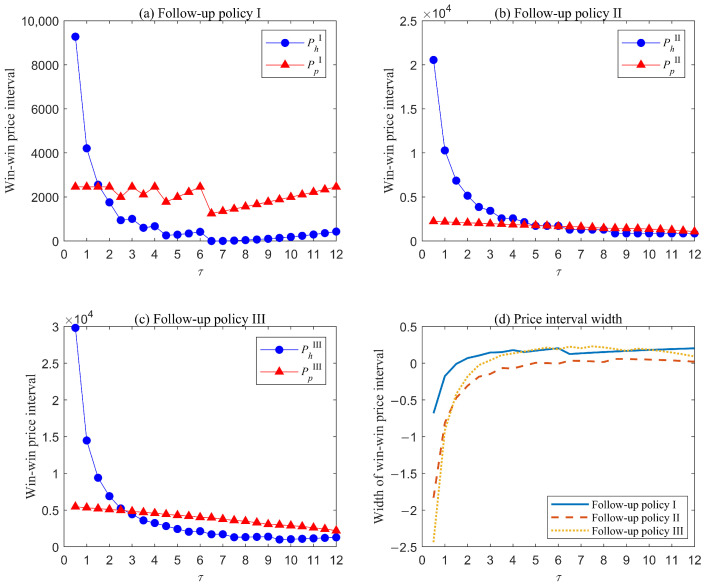
Results for the low-risk group. (**a**) Win–win price intervals under follow-up policy I; (**b**) win–win price intervals under follow-up policy II; (**c**) win–win price intervals under follow-up policy III; (**d**) price interval widths under the three follow-up policies.

We computed the win–win price intervals and widths of the three follow-up policies for each risk group separately under different values of τ. Figure 4 illustrates the results for the low-risk group. The following insights can be derived:

1. Overall, the minimum price hospitals will accept, $P_{h}^{X}$, under either follow-up policy decreases and remains nearly constant as τ increases. This occurs because the number of checkups provided under each policy decreases with τ, reducing the profit hospitals can obtain from follow-up services. Consequently, their acceptable price declines. Furthermore, when τ becomes sufficiently large, the number of checkups almost stabilizes, and the minimum price remains constant. Similarly, as the number of checkups decreases, the maximum price patients are willing to pay $P_{p}^{X}$ declines under either policy II or III, showing a more gradual trend. However, policy I exhibits a distinct pattern. This difference arises because the number of checkups under policy I is relatively low, meaning changes in τ cause a proportionally larger reduction in checkups. After τ≥6.5 months, the number of checkups during the warranty period stabilizes at one. At this point, patients become increasingly willing to pay for this single checkup to mitigate their AO risk and associated costs.
2. For follow-up policy I, after τ≥2, a win–win price interval emerges and the width of this interval increases slightly with τ. When τ≤6.5, the maximum price for patients fluctuates while the minimum price for hospitals reduces. However, once τ≥6.5, both the maximum price and the minimum price gradually rise. This occurs because, within these τ values, only one checkup occurs during the warranty period. When the checkup is scheduled near the end of the warranty period, AO risk and associated treatment cost during the warranty period increase, while those beyond the warranty decrease. Consequently, patients are more willing to pay for a checkup scheduled close to the warranty period, and hospitals can accept higher prices since the two parties bear AO treatment costs beyond and during the warranty period, respectively.
3. Under follow-up policy II, a win–win price interval emerges when τ≥6.5, with its width increasing rapidly and stabilizing almost completely as τ rises. Under this policy, patients bear the AO treatment cost during the post-warranty period. Consequently, AO risk and associated cost rise with τ, leading to a decline in the maximum price patients will pay. For hospitals, changes in τ primarily affect follow-up implementation cost, resulting in a gradual decrease in their minimum acceptable price. In addition, the interval width under this policy is minimal compared with that under policies I and III. This occurs because follow-up checkups scheduled too late fail to sufficiently reduce AO risk and cost, making patients reluctant to pay higher prices for these services.
4. Under follow-up policy III, win–win price intervals emerge when τ≥3. This policy enables patients to receive continuous follow-up services within the planning horizon, significantly reducing AO risk. Moreover, policy III reduces treatment costs for both hospitals during the warranty period and patients in the post-warranty period. The trends of maximum and minimum prices are similar to those under policy II, though both prices under policy III are consistently higher. This difference arises because policy III covers a longer time span, resulting in more follow-up checkups and higher associated costs.

**Figure 5:**
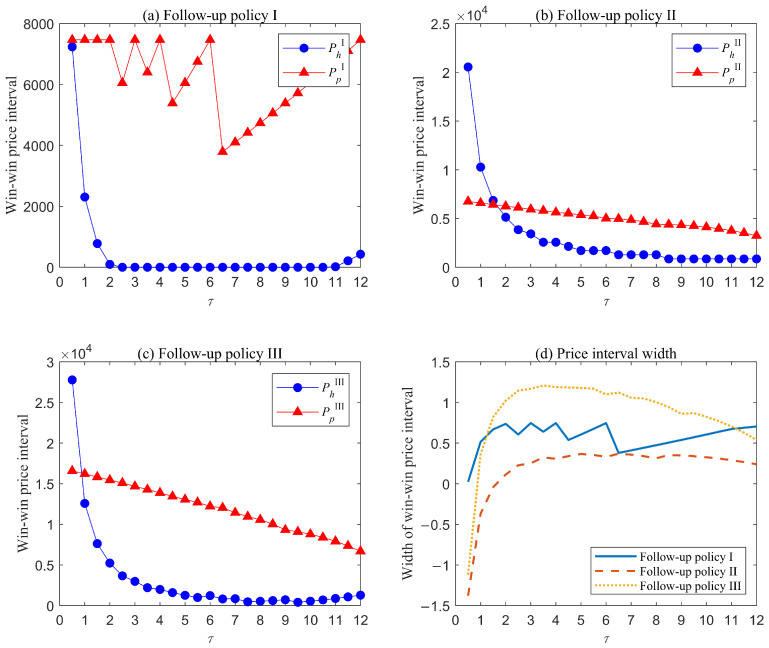
Results for the medium-risk group. (**a**) Win–win price intervals under follow-up policy I; (**b**) Win–win price intervals under follow-up policy II; (**c**) Win–win price intervals under follow-up policy III; (**d**) Price interval widths under three follow-up policies.

**Figure 6:**
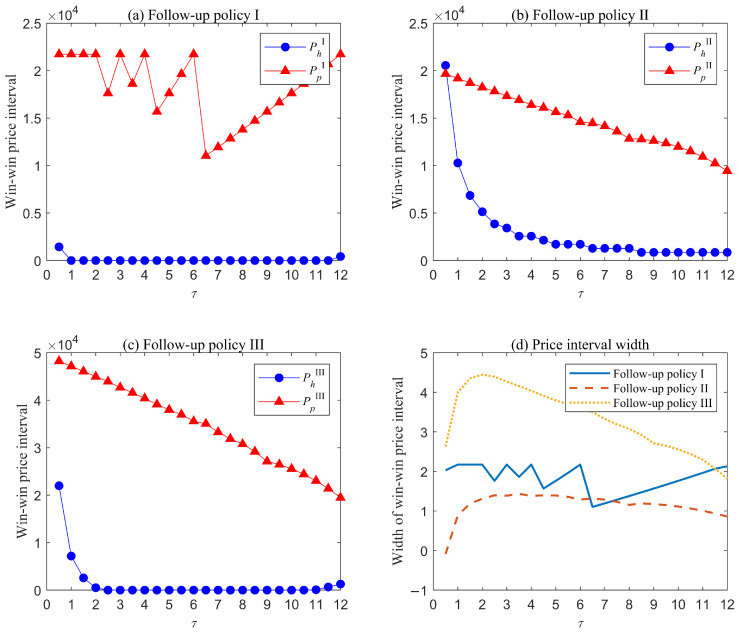
Results for the high-risk group. (**a**) Win–win price intervals under follow-up policy I; (**b**) Win–win price intervals under follow-up policy II; (**c**) Win–win price intervals under follow-up policy III; (**d**) Price interval widths under the three follow-up policies.

Figure 5 and Figure 6 present the results for the medium- and high-risk groups, respectively. For both groups, the overall trends in win–win price intervals and interval widths across different follow-up policies largely aligns with those observed in the low-risk group, though key differences emerge in the following aspects. First, win–win intervals appear earliest in the high-risk group, followed by the medium-risk group, and latest in the low-risk group, regardless of follow-up policy. For example, under follow-up policy II, win–win intervals in the three risks groups emerge when τ≥6.5, τ≥2, τ≥1, respectively. Second, the maximum price patients are willing to pay is highest for the high-risk group, intermediate for the medium-risk group, and lowest for the low-risk group. In contrast, the minimum price hospitals can accept follows an inverse pattern: highest for the low-risk group, intermediate for the medium-risk group, and lowest for the high-risk group, notably falling to zero in most cases. These patterns arise because higher-risk patients present more complex and severe conditions, resulting in elevated treatment cost during either warranty period or post-warranty period. Consequently, both patients and hospitals are willing to invest more in follow-up services, which can effectively reduce AO risk and associated cost.

### 3.3. Sensitivity Analysis

#### 3.3.1. Effects of the Age Reduction Factor θ

The age reduction factor θ measures the effectiveness of follow-up checkups in reducing AO risk. A smaller θ indicates greater checkup efficacy. Table 6 presents the win–win intervals for the three risk groups under different checkup age reduction factors, with τ fixed at 3 months. From the results in Table 6, we can observe that:

Low-risk group. Under follow-up policy I, win–win price intervals exist for age reduction factors $\theta =\left\{0.1,0.2,0.3,0.4\right\}$. In addition, these intervals narrow as θ increases, resulting in a higher minimum price threshold for hospitals and a lower maximum price threshold for patients. This occurs because less effective checkups increase hospitals’ expected number of AOs and costs during the warranty period, prompting them to demand higher prices for follow-up services to offset expenses. Conversely, patients will enter the post-warranty period with a higher AO risk and greater potential costs due to inefficient checkups, making them willing to pay less. Consequently, the maximum price threshold for patients decreases. For policy II, no win–win price interval exists at any θ value, indicating that post-warranty follow-up services provide no mutual advantage. Under policy III, a win–win price interval emerges only at θ=0.1, demonstrating that profitability requires highly effective checkups. When checkups are ineffective, AO risk reduction is insufficient in both warranty and post-warranty periods, yielding no benefits for either stakeholder.

Medium-risk group. Under follow-up policies I and III, win–win price intervals exist for all θ values. Similarly to the low-risk group, these intervals narrow as θ increases, for the reasons previously outlined. For policy II, win–win intervals exist when $\theta =\left\{0.1,0.2,0.3,0.4\right\}$, with all minimum prices for hospitals fixed at 3424 (equivalent to $n_{2}c_{f}$ shown in Equation (18)). The maximum prices for patients decrease as θ increases, consistent with observations under the other two policies. Compared to the low-risk group, win–win price intervals occur more frequently for the medium-risk group, because the higher AO risk enables even checkups with low effectiveness to sufficiently reduce expected AOs and associated cost. Consequently, both patients and hospitals show greater willingness to invest in follow-up services.

High-risk group. Win–win price intervals exist for all θ values under all follow-up policies. Consistent with the pattern observed in the low-risk group, the maximum prices for patients face decreases as θ increases. However, the trend for minimum prices differs slightly across policies. For policy I, the minimum price for hospitals is always zero, indicating that providing follow-up services to high-risk patients during the warranty period remains beneficial for hospitals regardless of checkup efficacy. For policy II, the minimum price for hospitals is consistently 3424, mirroring the medium-risk group scenario. This occurs because follow-up services are delivered after the warranty period, rendering them irrelevant to the cost decisions of hospitals. In addition, the downward trend in maximum prices for patients implies that win–win price intervals cease to exist when the price falls below 3424. For policy III, minimum prices start at zero when θ=0.1 or 0.2 but increase with θ. This reflects that for ineffective checkups, hospitals require fees to profit from follow-up services during the planning horizon.

#### 3.3.2. Effects of the Follow-Up Checkup Cost $c_{f}$

Table 7, Table 8 and Table 9 show the results of win–win price intervals under different follow-up checkup costs for the low-, medium- and high-risk groups, respectively, with τ fixed at 3 months. Looking at these results, we can see that:

For the low-risk group, win–win intervals consistently exist under follow-up policy I but do not emerge under policy II. Under policy III, these intervals exist specifically when the change in $c_{f}$ is {−30%, −20%, −10%, 0}. Within these existing intervals, the maximum price for patients remains constant under a given policy, while the minimum price for hospitals increases with $c_{f}$. This occurs because $c_{f}$ directly influences the hospital’s cost of implementing follow-up policies. As $c_{f}$ rises, hospitals require higher minimum prices to offset their increased implementation cost. Conversely, variations in $c_{f}$ do not affect the patient’s maximum price, as patients do not bear the cost of follow-up services. Consequently, the win–win price interval for follow-up services narrows as $c_{f}$ increases.

For the medium- and high-risk groups, win–win price intervals exist across all follow-up policies and varying $c_{f}$ levels. Similarly to the low-risk group, the maximum price for patients remains constant under any given policy. Regarding the minimum price for hospitals, it increases with $c_{f}$ under policy II for both groups and under policy III for the medium-risk group. Under the remaining policies and $c_{f}$ levels, the minimum price remains zero for these groups. However, even in these cases, the minimum price is projected to rise once $c_{f}$ becomes sufficiently high.

#### 3.3.3. Effects of the AO Treatment Cost $c_{kr}$

Table 10, Table 11 and Table 12 show the results of win–win price intervals under different AO treatment costs for the low-, medium- and high-risk groups, respectively, with τ fixed at 3 months. The following findings can be made:

For the low-risk group, win–win price intervals consistently exist under follow-up policy I but do not emerge under policy II. Under policy III, these intervals exist only when the change in $c_{1r}$ is {0, 10%, 20%, 30%}. Furthermore, these intervals widen as $c_{1r}$ increases for a given policy. Specifically, the minimum price for hospitals decreases, while the maximum price for patients increases. This widening occurs because rising $c_{1r}$ increases AO treatment costs for both parties: hospitals face higher treatment costs during the warranty period, and patients face higher treatment costs during the post-warranty period. To mitigate these rising costs, hospitals are incentivized to lower their minimum acceptable price to attract more follow-up service agreements. Patients become willing to pay a higher maximum price for follow-up services to avoid larger future expenses.

For the medium- and high-risk groups, win–win price intervals exist across all follow-up policies and values of $c_{kr}$. Within these intervals, the maximum price patients pay increases with $c_{kr}$, which is similar to the low-risk group. However, the minimum price hospitals can accept only decreases under policy III for the medium-risk group. Under other policies and across varying $c_{kr}$ levels, it remains largely stable. Specifically, under policy I, the minimum price starts at 202.41 for the medium-risk group but remains zero thereafter, while it is consistently zero for the high-risk group. This indicates hospitals’ willingness to provide low-price follow-up services during the warranty period, as AO treatment costs can be reduced to a large extent. Moreover, under policy II, the minimum price is calculated as $n_{2}c_{f}$, which is constant at 3424. This occurs because follow-up services are delivered post-warranty, where implementation costs are unaffected by $c_{kr}$.

#### 3.3.4. Effects of the Planning Horizon L

Table 13 shows win–win price intervals across different planning horizons L for the low-, medium- and high-risk groups, with τ fixed at 3 months. For the low-risk group, policy I consistently yields win–win intervals. The minimum price for hospitals remains fixed at 1002.65, while the maximum price for patients increases with L. This occurs because hospitals only cover treatment costs during the warranty period, which is unaffected by L, whereas patients bear post-warranty treatment costs that rise with longer horizons. Policy II generates win–win intervals only when L=5, indicating that follow-up services become mutually beneficial when the planning horizon is sufficiently long. Policy III produces win–win intervals when L is {3, 4, 5}, with both minimum and maximum prices rising with L. This is because follow-up services occur during the planning horizon, concurrently impacting hospitals’ implementation cost and patients’ treatment expenses. Similar patterns emerge for medium- and high-risk groups, with the key difference being that win–win price intervals exist across more policy–horizon combinations relative to the low-risk group.

## 4. Discussion

### 4.1. Principal Findings

Healthcare warranties transfer financial risk from patients to hospitals, incentivizing hospitals to provide long-term preventive services, such as follow-up policies, to chronic disease patients for cost control. However, the absence of an appropriate pricing mechanism impedes the practical implementation of these services, as they generate additional costs for hospitals. A feasible solution is to introduce patient copayments for follow-up services, making their pricing critically significant. To ensure mutual participation, pricing must balance two constraints. It should fall within the range that patients are willing to pay while exceeding hospitals’ minimum acceptable threshold. Against this backdrop, our paper investigates follow-up policy pricing for chronic diseases under healthcare warranties from a dual perspective. Accounting for patient heterogeneity, we employ a Cox frailty model to stratify patients into distinct risk groups and analyze pricing strategies per group. For each patient group, we evaluate three follow-up policies: periodic checkups during the warranty period, periodic checkups during the post-warranty period, and periodic checkups across the entire planning horizon. Using the virtual age method, we model hazard rate functions for each policy and derive total expected costs incurred by hospitals and patients via NHPP analysis. This enables identification of win–win price intervals, where upper bounds reflect maximum prices patients are willing to pay, and lower bounds represent hospitals’ minimum acceptable prices.

Applied to pediatric T1DM, our model yields actionable insights. First, win–win price intervals are more readily achievable for patients with higher risk, suggesting hospitals should prioritize these patients. Second, low-efficacy follow-up checkups narrow win–win price intervals. Therefore, hospitals must enhance checkup effectiveness in reducing AO risk to broaden pricing flexibility. Third, win–win price intervals narrow or remain stable as follow-up checkup cost increases, while widening as AO treatment cost rises. This indicates that hospitals must balance these two costs to optimize follow-up policy pricing. Fourth, both upper and lower price bounds increase with planning horizon length, making horizon selection crucial for feasible pricing, which can be further investigated in future research.

Conventional healthcare warranty (e.g., ProvenCare and PROMETHEUS) pricing typically consists of two main components: the average historical cost of providing follow-up services and AO treatments during the warranty period, plus an additional fee portion for AO treatments. Since both parts are calculated based on historical data, our proposed pricing method advances current knowledge in three key aspects. First, we incorporate the stochastic nature of AO occurrence and analyze their frequency and associated costs using stochastic processes, offering a significant advantage over static historical estimates. Second, our approach accounts for the influence of follow-up configuration on pricing, rather than simply averaging historical costs regardless of the number and frequency of follow-up checkups. Third, we consider not only the hospital’s perspective but also patients’ choices regarding follow-up policies, thereby enabling pricing that reflects both parties’ interests and promotes mutually beneficial outcomes. This dual perspective enhances joint and effective engagement in healthcare warranties and follow-up services.

### 4.2. Practical Implications

Our model will help hospitals effectively manage heterogeneous chronic patients and accurately determine reasonable pricing for follow-up services. First, by applying the patient stratification method proposed in our study, hospitals can categorize patients into different levels of AO risk and determine each patient’s specific risk level. Second, using the cost functions, hospitals can accurately estimate the costs incurred by both themselves and patients under various follow-up policies. Based on this, they can further derive win–win price intervals across different risk levels and types of follow-up policies. In addition, according to our principal findings, to obtain optimal and flexible intervals, hospitals are encouraged to improve the effectiveness of follow-up checkups in reducing AO risk, balance costs between follow-up checkups and AO treatment, and set appropriate planning horizons.

Our model also supports regulators (e.g., the government or insurers) in making decisions regarding reimbursement mechanisms and rates for follow-up policies for both hospitals and patients. Using the win–win price intervals generated by our model, regulators can further analyze the interrelationships between their own decisions and those of hospitals and patients. This enables the determination of optimal reimbursement mechanisms and rates that align with the interests and services of all three parties, thereby maximizing social welfare.

### 4.3. Limitations and Future Research

This study has several limitations on both model formulation and case study design, which offer avenues for future research.

Model formulation: Many promising extensions may be explored in future research based on our proposed model. First, the current model adopts a single cost perspective. One promising extension currently under consideration is formulating the follow-up policy pricing problem from a market perspective, incorporating factors such as the predicted sales volume of follow-up services and the utilities of both patients and hospitals. Additionally, this paper does not address scenarios where healthcare warranties can be renewed following hospitalizations due to AOs, which should be incorporated in future studies. Finally, this study investigates follow-up pricing with fixed components (e.g., follow-up frequency) under a healthcare warranty that also has fixed components (e.g., warranty period and planning horizon). Future research could treat these components as decision variables and explore the joint optimization of both the follow-up policy and the healthcare warranty structure.

Case study: Using pediatric T1DM as a case study, this paper validates the feasibility and applicability of the proposed model. However, some limitations exist in terms of data collection and analysis. First, the dataset is based on a relatively small sample size (37 patients with 67 AO treatment records and 149 follow-up records) from a single hospital, which may affect the statistical power and robustness of the results and limit the generalizability of the findings. Second, the study relies primarily on modeled outcomes and therefore requires further validation through empirical research. We are actively working to expand the dataset to mitigate these limitations. Third, as the study focuses on T1DM in children and adolescents, the selected risk factors (i.e., age, gender, puberty status, number of comorbidities, length of stay, and body mass index) were constrained by data availability. Health behaviors such as smoking and obesity were not incorporated, although they are recognized as significant factors influencing AO risk, follow-up frequency, and subsequent follow-up policy pricing [49,50,51]. Future research should aim to include these factors by expanding the scope to other chronic conditions, such as type 2 diabetes in adults.

## 5. Conclusions

This paper addressed the problem of adopting follow-up services for chronic diseases under healthcare warranties. A mathematical model was developed which offers a framework for the study of the opportunity provided by follow-up services from both patients’ and hospitals’ perspectives. The proposed framework evaluated three distinct follow-up policies featuring periodic checkups and modeled the respective hazard rate functions for each policy. For each policy, we quantified the total expected costs incurred by hospitals and patients across different risk levels. This enabled determination of the minimum price acceptable to hospitals for providing follow-up services and the maximum additional cost patients are willing to pay for such services. Consequently, win–win price intervals can be derived for each follow-up policy, tailored to heterogeneous patient risk profiles. Using T1DM as a case study, the results demonstrate that these price intervals depend critically on the risk level of patients, the follow-up policies, the average AO treatment cost, the age reduction factor and implementing cost of a checkup, as well as the length of the planning horizon. These insights facilitate strategic hospital decisions regarding follow-up arrangements for diverse risk cohorts and optimal price interval determination. This approach enhances medical resource allocation, improves chronic disease management efficacy, and ultimately enables cost control.

## Figures and Tables

**Table 2:** Hazard rate functions under different follow-up policies.

Follow-Up Policy I	Hazard Rate Function
$\left[0\right.,\left.W\right)$	$\lambda_{W}^{I}\left(t|h\right)=\left\{\begin{array}{c} \lambda \left(v_{i-1}+t-t_{i-1}|h\right),\\ \lambda \left(v_{n_{1}}+t-t_{n_{1}}|h\right), \end{array}\right.\begin{array}{c} t_{i-1}\leq t<t_{i},i=1,2,\cdots ,n_{1}\\ t_{n_{1}}\leq t<W \end{array}$
$\left[W\right.,\left.L\right)$	$\lambda_{PW}^{I}\left(t|h\right)=\lambda \left(v_{n_{1}}+t-t_{n_{1}}|h\right),W\leq t\leq L$
Follow-up policy II	
$\left[0\right.,\left.W\right)$	$\lambda_{W}^{II}\left(t|h\right)=\lambda \left(t|h\right),0\leq t<W$
$\left[W\right.,\left.L\right)$	$\lambda_{PW}^{II}\left(t|h\right)=\left\{\begin{array}{c} \lambda \left(t|h\right),\\ \lambda \left(v_{i-1}+t-t_{i-1}|h\right),\\ \lambda \left(v_{n_{2}}+t-t_{n_{2}}|h\right), \end{array}\right.\begin{array}{c} W\leq t<t_{1}\\ t_{i-1}\leq t<t_{i},i=2,\cdots ,n_{2}\\ t_{n_{2}}\leq t<L \end{array}$
Follow-up policy III	
$\left[0\right.,\left.W\right)$	$\lambda_{W}^{III}\left(t|h\right)=\left\{\begin{array}{c} \lambda \left(v_{i-1}+t-t_{i-1}|h\right),\\ \lambda \left(v_{n_{4}}+t-t_{n_{4}}|h\right), \end{array}\right.\begin{array}{c} t_{i-1}\leq t<t_{i},i=1,2,\cdots ,n_{4}\\ t_{n_{4}}\leq t<W \end{array}$
$\left[W\right.,\left.L\right)$	$\lambda_{PW}^{III}\left(t|h\right)=\left\{\begin{array}{c} \begin{array}{c} \lambda \left(v_{n_{4}}+t-t_{n_{4}}|h\right),\\ \lambda \left(v_{i-1}+t-t_{i-1}|h\right), \end{array}\\ \lambda \left(v_{n_{3}}+t-t_{n_{3}}|h\right), \end{array}\right.\begin{array}{c} \begin{array}{c} W\leq t<t_{n_{4}+1}\\ t_{i-1}\leq t<t_{i},i=\left(n_{4}+1\right),\left(n_{4}+2\right),\cdots ,n_{3} \end{array}\\ t_{n_{3}}\leq t<L \end{array}$

**Table 3:** Number of follow-up checkups within different time periods.

Number	Definition	Calculation
$n_{1}$	Number of checkups under follow-up policy I within $\left(0\right.,\left.W\right]$	$n_{1}=\left\lfloor \frac{W}{\tau }\right\rfloor$ ^1^
$n_{2}$	Number of checkups under follow-up policy II within $\left(W\right.,\left.L\right]$	$n_{2}=\left\lfloor \frac{L-W}{\tau }\right\rfloor$
$n_{3},n_{4}$	Number of checkups under follow-up policy III within $\left(0\right.,\left.L\right]$ and $\left(0\right.,\left.W\right]$	$n_{3}=\left\lfloor \frac{L}{\tau }\right\rfloor$ $n_{4}=\left\lfloor \frac{W}{\tau }\right\rfloor$

^1^ $\left\lfloor X\right\rfloor$: floor function of X.

**Table 4:** Risk groups and average treatment cost for AOs.

k	Risk Group	$\left[h_{k-1},h_{k}\right)$	$c_{kr}$
1	Low-risk	$\left[0.0449,0.1331\right)$	6520
2	Medium-risk	$\left[0.1331,0.2300\right)$	10,200
3	High-risk	$\left[0.2300,0.7182\right]$	15,580

**Table 5:** Parameters for the case study.

$c_{f}$	θ	W	L
428	0.1	1 year	3 years

**Table 6:** Win–win price intervals under different age reduction factors.

Low-risk group	Follow-up policy I	Follow-up policy II	Follow-up policy III
θ=0.1	[1002.65, 2456.23]	∅	[4426.65, 4828.20]
θ=0.2	[1102.65, 2143.14]	∅	∅
θ=0.3	[1193.66, 1844.86]	∅	∅
θ=0.4	[1278.42, 1558.31]	∅	∅
θ=0.5	∅	∅	∅
Medium-risk group	Follow-up policy I	Follow-up policy II	Follow-up policy III
θ=0.1	[0, 7467.43]	[3424, 5947.77]	[2979.45, 14,678.69]
θ=0.2	[0, 6515.56]	[3424, 5217.50]	[3283.47, 12,159.88]
θ=0.3	[136.14, 5609.04]	[3424, 4509.20]	[3560.14, 9960.56]
θ=0.4	[393.83, 4737.58]	[3424, 3820.32]	[3817.83, 7961.82]
θ=0.5	[637.07, 3895.02]	∅	[4061.07, 6106.14]
High-risk group	Follow-up policy I	Follow-up policy II	Follow-up policy III
θ=0.1	[0, 21,724.42]	[3424, 17,303.39]	[0, 42,703.55]
θ=0.2	[0, 18,955.20]	[3424, 15,178.85]	[0, 35,375.77]
θ=0.3	[0, 16,317.94]	[3424, 13,118.27]	[551.49, 28,977.48]
θ=0.4	[0, 13,782.66]	[3424, 11,114.14]	[1301.16, 23,162.69]
θ=0.5	[0, 11,331.48]	[3424, 9106.42]	[2008.79, 17,764.11]

**Table 7:** Win–win price intervals under different $c_{f}$ (low-risk group).

$\mathrm{Percentage}\;\mathrm{of}\;\mathrm{Change}\;(c_{f}$)	Follow-Up Policy I	Follow-Up Policy II	Follow-Up Policy III
−30%	[489.05, 2456.23]	∅	[2885.85, 4828.20]
−20%	[660.25, 2456.23]	∅	[3399.45, 4828.20]
−10%	[831.45, 2456.23]	∅	[3913.05, 4828.20]
0	[1002.65, 2456.23]	∅	[4426.65, 4828.20]
10%	[1173.85, 2456.23]	∅	∅
20%	[1345.05, 2456.23]	∅	∅
30%	[1516.25, 2456.23]	∅	∅

**Table 8:** Win–win price intervals under different $c_{f}$ (medium-risk group).

$\mathrm{Percentage}\;\mathrm{of}\;\mathrm{Change}\;(c_{f}$)	Follow-Up Policy I	Follow-Up Policy II	Follow-Up Policy III
−30%	[0, 7467.43]	[2396.80, 5947.77]	[1438.65, 14,678.69]
−20%	[0, 7467.43]	[2739.20, 5947.77]	[1952.25, 14,678.69]
−10%	[0, 7467.43]	[3081.60, 5947.77]	[2465.85, 14,678.69]
0	[0, 7467.43]	[3424, 5947.77]	[2979.45, 14,678.69]
10%	[0, 7467.43]	[3766.40, 5947.77]	[3493.05, 14,678.69]
20%	[0, 7467.43]	[4108.80, 5947.77]	[4006.65, 14,678.69]
30%	[69.05, 7467.43]	[4451.20, 5947.77]	[4520.25, 14,678.69]

**Table 9:** Win–win price intervals under different $c_{f}$ (high-risk group).

$\mathrm{Percentage}\;\mathrm{of}\;\mathrm{Change}\;(c_{f}$)	Follow-Up Policy I	Follow-Up Policy II	Follow-Up Policy III
−30%	[0, 21,724.42]	[2396.80, 17,303.39]	[0, 42,703.55]
−20%	[0, 21,724.42]	[2739.20, 17,303.39]	[0, 42,703.55]
−10%	[0, 21,724.42]	[3081.60, 17,303.39]	[0, 42,703.55]
0	[0, 21,724.42]	[3424, 17,303.39]	[0, 42,703.55]
10%	[0, 21,724.42]	[3766.40, 17,303.39]	[0, 42,703.55]
20%	[0, 21,724.42]	[4108.80, 17,303.39]	[0, 42,703.55]
30%	[0, 21,724.42]	[4451.20, 17,303.39]	[402.92, 42,703.55]

**Table 10:** Win–win price intervals under different $c_{1r}$.

$\mathrm{Percentage}\;\mathrm{of}\;\mathrm{Change}\;(c_{1r}$)	Follow-Up Policy I	Follow-Up Policy II	Follow-Up Policy III
−30%	[1215.46, 1719.36]	∅	∅
−20%	[1144.52, 1964.99]	∅	∅
−10%	[1073.59, 2210.61]	∅	∅
0	[1002.65, 2456.23]	∅	[4426.65, 4828.20]
10%	[931.72, 2701.86]	∅	[4355.72, 5311.02]
20%	[860.79, 2947.48]	∅	[4284.79, 5793.84]
30%	[790.83, 3189.71]	∅	[4214.83, 6270]

**Table 11:** Win–win price intervals under different $c_{2r}$.

$\mathrm{Percentage}\;\mathrm{of}\;\mathrm{Change}\;(c_{2r}$)	Follow-Up Policy I	Follow-Up Policy II	Follow-Up Policy III
−30%	[202.41, 5227.20]	[3424, 4163.44]	[3626.41, 10,275.08]
−20%	[0, 5973.95]	[3424, 4758.22]	[3410.76, 11,742.95]
−10%	[0, 6720.69]	[3424, 5353]	[3195.10, 13,210.82]
0	[0, 7467.43]	[3424, 5947.77]	[2979.45, 14,678.69]
10%	[0, 8214.18]	[3424, 6542.55]	[2763.79, 16,146.56]
20%	[0, 8960.92]	[3424, 7137.33]	[2548.14, 17,614.43]
30%	[0, 9707.67]	[3424, 7732.11]	[2332.48, 19,082.30]

**Table 12:** Win–win price intervals under different $c_{3r}$.

$\mathrm{Percentage}\;\mathrm{of}\;\mathrm{Change}\;(c_{3r}$)	Follow-Up Policy I	Follow-Up Policy II	Follow-Up Policy III
−30%	[0, 15,207.09]	[3424, 12,112.37]	[744.28, 29,892.48]
−20%	[0, 17,379.53]	[3424, 13,842.71]	[116.89, 34162.84]
−10%	[0, 19,551.97]	[3424, 15,573.05]	[0, 38,433.19]
0	[0, 21,724.42]	[3424, 17,303.39]	[0, 42,703.55]
10%	[0, 23,896.86]	[3424, 19,033.73]	[0, 46,973.90]
20%	[0, 26,069.30]	[3424, 20,764.07]	[0, 51,244.26]
30%	[0, 28,241.74]	[3424, 22,494.40]	[0, 55,514.61]

**Table 13:** Win–win price intervals under different planning horizons.

Low-risk group	Follow-up policy I	Follow-up policy II	Follow-up policy III
L=2	[1002.65, 1388.35]	∅	∅
L=3	[1002.65, 2456.23]	∅	[4426.65, 4828.20]
L=4	[1002.65, 3383.50]	∅	[6138.65, 8664.37]
L=5	[1002.65, 4223.42]	[6848, 7573.04]	[7850.65, 13,247.72]
Medium-risk group	Follow-up policy I	Follow-up policy II	Follow-up policy III
L=2	[0, 4220.86]	∅	[1267.45, 5542]
L=3	[0, 7467.43]	[3424, 5947.77]	[2979.45, 14,678.69]
L=4	[0, 10,286.52]	[5136, 13,263.90]	[4691.45, 26,341.40]
L=5	[0, 12,840.02]	[6848, 23,023.55]	[6403.45, 40,274.40]
High-risk group	Follow-up policy I	Follow-up policy II	Follow-up policy III
L=2	[0, 12,279.42]	[1712, 3975.36]	[0, 16,122.90]
L=3	[0, 21,724.42]	[3424, 17,303.39]	[0, 42,703.55]
L=4	[0, 29,925.76]	[5136, 38,587.61]	[574.12, 76,632.95]
L=5	[0, 37,354.47]	[6848, 66,980.58]	[2286.12, 117,170.03]

## Data Availability

The data presented in this study are available on request from the corresponding author due to privacy.

## Abbreviations

The following abbreviations are used in this manuscript:

AOs	Adverse Outcomes
CMS	Centers for Medicare & Medicaid Services
MDPs	Markov Decision Processes
BPCI	Bundled Payments for Care Improvement
FFS	Fee-For-Service
T1DM	Type 1 Diabetes Mellitus
CDF	Cumulative Distribution Function
PDF	Probability Density Function
NHPP	Non-Homogeneous Poisson Process

## Appendix A

### Appendix A.1. Proof of Equation (15)

$$C_{py}^{I}\leq C_{pn}^{I}⟺P_{k}+c_{kr}\int_{h_{k-1}}^{h_{k}}\int_{W}^{L}\lambda_{PW}^{I}\left(t|h\right)dtdG\left(h\right)\leq c_{kr}\int_{h_{k-1}}^{h_{k}}\int_{W}^{L}\lambda \left(t|h\right)dtdG\left(h\right)\;\Rightarrow P_{k}\leq c_{kr}\left[\int_{h_{k-1}}^{h_{k}}\int_{W}^{L}\lambda \left(t|h\right)dtdG\left(h\right)-\int_{h_{k-1}}^{h_{k}}\int_{W}^{L}\lambda_{PW}^{I}\left(t|h\right)dtdG\left(h\right)\right]=c_{kr}\phi \left(h_{k}\right)\int_{W}^{L}\left[\lambda_{0}\left(t\right)-\lambda_{0}\left(v_{n_{1}}+t-t_{n_{1}}\right)\right]dt=P_{p}^{I}.$$

### Appendix A.2. Proof of Equation (16)

$$C_{hy}^{I}\leq C_{hn}^{I}⟺c_{kr}\int_{h_{k-1}}^{h_{k}}\int_{0}^{W}\lambda_{W}^{I}\left(t|h\right)dtdG\left(h\right)+n_{1}c_{f}-P_{k}\leq c_{kr}\int_{h_{k-1}}^{h_{k}}\int_{0}^{W}\lambda \left(t|h\right)dtdG\left(h\right)\;\Rightarrow P_{k}\geq c_{kr}\left[\int_{h_{k-1}}^{h_{k}}\int_{0}^{W}\lambda_{W}^{I}\left(t|h\right)dtdG\left(h\right)-\int_{h_{k-1}}^{h_{k}}\int_{0}^{W}\lambda \left(t|h\right)dtdG\left(h\right)\right]+n_{1}c_{f}=c_{kr}\phi \left(h_{k}\right)\left[\sum_{i=1}^{n_{1}}\int_{t_{i-1}}^{t_{i}}\lambda_{0}\left(v_{i-1}+t-t_{i-1}\right)dt+\int_{t_{n_{1}}}^{W}\lambda_{0}\left(v_{n_{1}}+t-t_{n_{1}}\right)dt-\int_{0}^{W}\lambda_{0}\left(t\right)dt\right]+n_{1}c_{f}=P_{h}^{I}.$$
